# Toward a sociology of evolution in the Anthropocene–Shared intentionality and cooperation through understanding minds

**DOI:** 10.3389/fsoc.2022.1079879

**Published:** 2022-12-15

**Authors:** Ludger Pries

**Affiliations:** Faculty of Social Science, Ruhr-University Bochum, Bochum, Germany

**Keywords:** evolution science, sociology of evolution, Anthropocene, nature-culture-technology, cooperation

## Abstract

Sociology has a long tradition of diagnosing contemporary societies, but little theoretical and empirical instruments for analyzing the long-term evolution of human coexistence. This goes hand in hand with a bias to disregard insights of evolutionary theory and research. The main argument here to develop is that a sociology of evolution should enter at the core of our discipline. This becomes even more important in the era of the Anthropocene as a new geo-chronological period of the planet's evolution that is characterized by substantial human influencing of planetary ecological mechanisms and could be found in earth sediments. If human intervention in the planet has reached such a scale that its future fate is no longer shaped mainly by natural cosmological laws, but by human intervention, then sociology has to broaden its temporal and substantive perspective; it should reflect more explicitly on the relationship between nature, culture, and technology. In what follows, we plead for giving evolutionary sociology, especially the long-term evolution of human coexistence between nature and culture, a greater place in sociology. To this end, we address three points. First, we ask why sociology is not concerned with the co-evolution of other creatures, but almost exclusively focused on the development and social change of humans over the short period of the last few centuries. Second, we argue that, with respect to the nature-culture relationship, sociology has essentially followed a questionable scientific division of labor, according to which the natural sciences deal with natural phenomena and sociology with sociocultural phenomena. Finally, we address the debate on the Anthropocene and distinguish between two ways of responding to the challenges it poses, namely with more technology or with more culture.

## Introduction

Sociology has a long tradition of *diagnosing* contemporary societies. Since its beginnings, it has distinguished societies characterized by mechanical or organic solidarity, by the prevalence of *communitization* or *socialization* (Vergemeinschaftung and Vergesellschaftung, Weber, 1978), by simple or reflexive modernization (Beck et al., 1994), as industrial, postindustrial, service, or knowledge societies (Sennet, 2006). With the beginning of the new century, a new term, the Anthropocene, entered the sciences. This is meant to characterize a new geo-chronological period of the planet's evolution. In the geosciences, a change in the planet's sediments is diagnosed for the middle of the 20th century, indexing concentrated radioactivity from atomic bombs and profound changes in the atmosphere. A “strong acceleration” of many factors (world population, fossil energy consumption, use of artificial fertilizers, etc.) is diagnosed, which are man-made and produce geo-planetary impacts (Steffen et al., 2015, p. 84). According to the Anthropocene thesis, human intervention in the planet has reached such a scale that its future fate is no longer shaped mainly by natural cosmological laws, but by human intervention.

In light of this, we have to broaden our temporal and substantive perspective in social sciences. Time diagnoses that refer to a few decades or centuries are not sufficient. Besides social, cultural, economic and political aspects, we have to include natural and evolutionary dimensions. We should reflect more explicitly on the relationship between nature, culture, and technology. In what follows, we plead for giving evolutionary sociology, especially the long-term evolution of human coexistence between nature and culture, a greater place in social sciences, especially in sociology. To this end, we address three points. First, we ask why sociology is not concerned with the co-evolution of other creatures, but almost exclusively focused on the development and social change of humans over the short period of the last few centuries. Second, we argue that, with respect to the nature-culture relationship, sociology has essentially followed a questionable scientific division of labor, according to which the natural sciences deal with natural phenomena and sociology with sociocultural phenomena. Finally, we address the debate on the Anthropocene and distinguish between two ways of responding to the challenges it poses, namely with more technology or with more culture.

## (Why) does sociology have a problem with evolution?

For a long time, in social sciences a positivist idea similar to classic evolutionary theory prevailed. According to this, knowledge is produced in a cumulative way and theories assert themselves or die according to the criteria of truth and rationality. Correspondingly, natural and social reality is explained better and better in a linear way and by the evolutionary mechanism of trial and error. Today we know that the development of knowledge is more complex, that there are paradigms, and that science is organized in social contexts of (gendered, postcolonial, South-North and West-East) interests and power relations, of striving for recognition and success (Knorr-Cetina, 1981; Leibowitz, 2017; Crawford et al., 2021). Thus, knowledge development is partly cumulative and–especially in the humanities, cultural studies, and social sciences–works in paradigm cycles and asymmetric power relations. This is also true for evolutionary research.

In the social sciences, philosophical anthropology in particular asked what special features distinguish humans from other animals. It pursued this question less by empirical analysis than by “introspection,” by reflection on one's own experiences of life and perception. In his book on the “special position of man in the universe” Max Scheler wrote (in a time-diagnostic perspective): “We are the first age in approximately ten thousand years of history, in which man has become completely and restless problematic: in which he no longer knows what he is; but at the same time he knows that he does not know” (Scheler, 1927, p. 120). Already Scheler emphasized the essence of humans compared to all other animals: as hominids we can reflexively ask ourselves questions about our own existence. Neverless, as fruitful as philosophical anthropology may be for the understanding of human evolution: it cannot have knowledge of the (possible) introspection of other living beings and is unable to produce intersubjectively verifiable empirical evidence.

As sociology was for a long time little concerned with evolutionary research, biology was perceived as the only discipline for the empirical analysis of the evolution of nature, including plants and all living things. With respect to the evolution of humans, such a claim is problematic because here nature and culture are closely intertwined and sociology as well as other sciences have specific competencies for cultural analyses. Moreover, in the popular reception of evolutionary theory, a highly reduced version of Darwinism prevailed. According to this, evolution is a mechanism of random mutations (*trial and error*) and the selection of the living beings best adapted to changing environmental conditions (*survival of the fittest*).

Such a truncated representation of evolution theory could legitimize modern capitalism as a natural-evolutionary way of human coexistence: technical innovations work like mutations, market competition filters the survival of the fittest. Such an abbreviated and biased evolutionary narrative can be found in Herbert Spencer's Social Darwinism and in the racial theories of colonialism and fascism. All of this probably contributed to sociology's skepticism about evolutionary research. However, our discipline largely accepted an inappropriate division of labor according to which biology and other natural sciences are responsible for natural phenomena and sociology for sociocultural phenomena. Sociology established itself almost in parallel with Darwinism. Darwin published his main work in 1858, when Karl Marx issued already foundations of critical social theory. Toward the end of the 19th century, the classical publications of the founders of sociology such as Auguste Comte, William Edward Du Bois, Emile Durkheim, Vilfredo Pareto, Herbert Spencer and Max Weber began.

In order to establish itself as a genuine scientific discipline, sociology sought to explain the social exclusively through the social. Émile Durkheim proposed that the object of study of sociology should be the social facts, “*le fait social total*” that could be explained exclusively by the social. For Max Weber, too, the core of sociology was the social when he defines it as a “science which seeks to understand social action in an interpretative way and thereby to explain it causally in its course and effects” (Weber, 1978, p. 1). It is interesting to note that Weber himself suffered from serious illnesses that also led him to reflect in his private correspondence on the relationship between nature or the body and his social life. However, he never included the relationship between nature and culture more explicitly in scholarly publications (Radkau, 2005).

The exclusion of nature from sociological reflection was taken to the extreme in systems theory. It is an irony of history that Niklas Luhmann drew his ideas of autopoiesis, for example, from biology and Humberto Maturana's theory of natural systems, but nevertheless continued the tendency to explain the social exclusively by the social (Lipp, 1987). Skepticism about an integral view of nature and culture may also have been reinforced in sociology by the fact that many attempts have been made in biology to apply biological explanations mechanistically to all kinds of social behavior and social action. In much of biology, there is a functionalist tendency to explain every phenomenon of symbiosis, proto-social behavior, or altruism in animals exclusively by natural mechanisms such as the “selfishness of genes” without any reference to culture.^1^ This may also clarify part of reservations in sociology about (misunderstood or biologistic) evolutionary research.

A final aspect that can explain - but not scientifically legitimize - the divide in sociology between the consideration of the natural and the social, between nature and culture, is the long-accepted notion that man is the “crown of creation” because of his special abilities. In sociology as well as biology, only man was attributed emotions, culture, grief, and so on. The sciences as a whole assigned culture, or rational and social control of action, to man and nature, or instinct-driven behavior, to other living beings. This claimed clear distinction between culture and nature was then also supposed to legitimize the power of humans to use the “lower creatures.” In the slavery of antiquity, in colonialism and in National Socialism, even certain groups of people were defined as “inferior by nature” and from this unlimited rights of disposal were derived toward them.^2^

In the 21st century, the separate dedication of scientific disciplines either to nature or to culture as well as non-cooperation between sociology and biology become problematic. Culture as all knowledge that is not genetically transferred, but learned and passed on intergenerationally through social interaction is increasingly addressed in biology. Many studies from biology and behavioral sciences show that not only the coexistence of humans, but also of many animal species is culturally differentiated. The specific forms of communication of dolphins or primates are learned during socialization; primate groups have their own dialects and food preferences that evolve in interaction with changing environments (Richerson and Boyd, 2005). Animal species do not merely adapt reactively to existing environmental conditions, but actively create their niches for life; humans create their “cognitive niche” (Pinker, 2010; Schuppli et al., 2016). In the Anthropocene, humans have made the entire planet their “niche” through agriculture, industrial production, buildings, the Internet, and social media. “Nature” is already saturated by human activities; it is *cultural nature*. Other animals also create their own niches actively (Turner and Maryanski, 2019, p. 94f).

With the analytical capabilities of paleogenetics, parts of the long history of humans can be reconstructed, in particular the migrations and encounters between different human species from *Homo heidelbergensis, Homo floresiensis, Homo denisovanes* to *Homo sapiens*. Geomorphology provides new insights into the impact of human activities on the evolution of the planet. Krause and Trappe (2021) summarize results of studies on evolutionary anthropology and paleogenetics and show that there was direct contact and genetic mixing between *Homo neandertalensis* and *Homo sapiens* over tens of thousands of years. Clearly, this was not simply a deadly fighting between and within species, but an interrelationship of competition, coexistence, and cooperation.

Today, we also know more about the mechanism of epigenesis as a bridge between genetically programmed mechanisms and learning in natural environments. Epigenetics explores how certain gene segments can also be altered in the status of their activation by environmental influences. For example, plants that have survived a severe drought store this episode through chemical activations in corresponding gene segments. When the causative environmental influences occur again, they can adapt more quickly. Thus, it is not the sequence of base pairs in the DNA structure of the genes that is directly changed, but the activation status of specific gene segments is stored as in a memory; this epigenetic change based on individual experience can be passed on inter-generationally: “Epigenesis, originally a biological concept, means the development of an organism under the joint influence of heredity and environment” (Wilson, 1999, p. 210). Similarly, animals and humans can store existential experiences epigenetically: not by changing gene sequences, but chemical activation changes in specific gene regions. Through this mechanism, plants, animals and humans have learning mechanisms beyond the purely genetic and the cultural-communicative transmission of experience (see e.g., Gilbert et al., 2010; Negri and Jablonka, 2016; Meloni, 2019).

Another finding of the last decades is the empirical evidence that animals can develop differentiated feelings, which we used to attribute only to humans. Charles Darwin had already observed this in his dog. But today we know that elephants, for example, feel emotions such as grief and create graveyards (Moss and Colbeck, 1992). It has also been proven that animals, like humans, can practice altruism and selfless cooperation. This contradicts a truncated Darwinism that explains everything natural in terms of the mechanism of existential fighting between individuals, populations, and species. Altruism and symbiosis are keywords of recent research and refer to mechanisms of interdependent cooperation between species. As will be shown in the following, humans are characterized by the ability to cooperation through comprehending minds. In sum, more recent evolutionary research forces all disciplines, including sociology, to fundamentally rethink the separation between nature and culture.

## Nature and culture in human development

In an evolutionary perspective, nature and culture can be distinguished by the respective mechanism of intergenerational transmission of information and knowledge. By *nature* we mean all those traits, abilities and behaviors that are genetically inherited from generation to generation. As *culture* we refer to that part of the characteristics, abilities and behavioral regularities that are passed on intergenerationally through learning. “Culture works on the basis of various kinds of transmission systems [...], which collectively provide humans with a second, non-genetic 'knowledge-carrying' inheritance system.” (Laland et al., 2000, p. 132). The human ape genus *Danuvius guggenmosi* emerged about ten million years ago; the history of *Homo sapiens* accounts for <5 percent of this period (Krause and Trappe, 2021, p. 60ff). The transmission of evolutionarily relevant information was initially predominantly through genes and then increasingly through learning. Intergenerational knowledge transmission through culture and learning increased only in the last two to three million years (when the use of stones as tools began), expanded considerably in the last 800,000 years (through the use of fire), and just exploded in the last 15,000 years, when sedentary life and agriculture began (Krause and Trappe, 2021, p. 178ff).

This human evolution took place in the context of long-term climate changes, each of which redefined the conditions of life on all continents and the possibilities of passing through certain migration corridors. In the long period of the last ten million years, the average global temperature decreased from about 20–14 degrees Celsius. At the same time, the amplitudes of temperature fluctuations increased in the “short” period of decades to millennia.^3^ As a result, rapid adaptability gained importance as an evolutionary criterion. One million years ago, the passage from Africa to Arabia was opened by a route in the south of the Arabian Peninsula (where, due to the sea level, the distance to be covered by ship was much shorter than before and after) and by a northern route through today's Palestine and Israel (where, due to the climatic conditions, a passage was possible in exactly this period; Krause and Trappe, 2021, p. 86ff). About 15,000 years ago, the land passage through the Bering Strait was opened, leading to the settlement of the Americas (Krause and Trappe, 2021, p. 135).

In addition to the general evolutionary mechanisms of (passive) environmental adaptation, migration, natural symbioses, and (active) niche construction, only humans developed the capabilities of *complex sociocultural communication and cooperation through understanding minds*, which enabled a speed of intergenerational knowledge transfer and complexity of social coexistence not achieved by other living beings. It is precisely here that sociology can make crucial contributions to the analysis of human evolution. This will be sketched out in the following in light of the classical Darwinian model, Neo-Darwinism, and the so-called Extended Evolutionary Synthesis (EES).

The classical Darwinian model focuses on *natural* mechanisms of evolution. The transfer of information is organized by genes in such a way that only those creatures, populations and species survive that are best adapted to their environment and can leave behind successful offspring. This “natural selection” is based on random mutations and the blind process of trial and error. Instincts are understood as natural patterns of behavior. Darwin himself argued in a much more sophisticated way (Pries, 2021, chap. 2). But a truncated Darwinism dominated the general understanding of evolution until the end of the 20th century and was taught in schools and universities (except in highly specialized courses; see Table 1).

**Table 1:** Three conceptual stages of evolution.

		**Darwinism**	**Neo-darwinism/EES**	**Sociocultural model**
Nature (genetic transmission)	Change	Contingent/blind mutation (trial and error) in individuals, competition	Contingent/blind mutation, competition, sexuality, gene expression, epigenetics.	Mutation + symbiosis + intra-/inter-group division of labor, competition, conflict.
	Stabilization	Natural selection through competition/survival of the fittest; instincts	Natural selection, epigenetics, sociality.	Linguistic ability, cognitive competence, environmental and niche production, ‘instinctual stumps'.
Culture (symb.-comm. sharing)	Change		*Cultural group selection* through competition and struggle, *cultural trial-and-error*.	Understanding of meaning, shared/comprehensive intentionality, social learning and work-sharing, creativity.
	Stabilization		Survival of the culturally fittest, niche production, cultural communication.	Cooperation through understanding minds, institutions, technology, socialization, life course, innovation.

Source: Author's own elaboration.

At the same time, criticism of Darwin's work had been developing since the 1880's. Neo-Darwinism (Edgar Wallace, August Weismann) showed that phenotypic variability arises primarily from bisexual insemination and that traits acquired in the individual life course are not inherited genetically. Then, in the 20th century, EES emphasized that selection alone could not produce innovation in evolutionary terms. It emphasized the mechanisms of epigenetics and gene expression mentioned earlier. In (embryonic) ontogeny, it is not the total number of all gene sequences that is at play; rather, certain gene segments serve as switches that activate other areas of the genome. In humans, of the approximately two-meter-long genome in each stem cell, less than five percent is relevant to a person's ontogenetic development. Complex organs develop in an embryo as a result of certain areas in the genes being switched on and off at a certain point in time: “The diversity of species in the context of basic blueprints is based primarily on variations in the switching on and off of genes and not on mutations” (Neuweiler, 2008, p. 51).

Especially since the second half of the 20th century, the concepts of creative niche construction and *cultural* group selection have been developed. Social subgroups seek and create specific *cultural niches* (Boyd and Richerson, 1985; Scott, 2020, p. 51ff, 54). “In our view, the capacity of populations of organisms to modify their selective environment through niche construction, and the fact that many of these changes persist for multiple generations, demand an adjustment in our understanding of the evolutionary dynamic, because they suggest that a description of evolutionary change relative only to independent environments is rather restrictive” (Laland et al., 2000, p. 135). Evolution is not contingent mutation, passive environmental adaptation, and selection of the fighting fittest, but rather certain subunits of a species create their own habitats based on a logic of cultural learning and intergeneric transmission. For humans, the development of new hunting, planting, and harvesting techniques, as well as the migration of populations from one place to another, are examples of active intervention in and even direct shaping of their environment and living conditions (Scott, 2020). Biologists Richerson and Boyd (2005) speak of the “*survival of the cultural fittest.”* Evolution is above all creativity and the ability to innovate in the face of dynamic environments.

Since the 21st century, evolutionary research has increasingly included the mechanisms of symbiosis and cooperation. The biologist Lynn Margulis had already developed the theory of endosymbionts in the 1960's. According to this theory, all eukaryotes, i.e., all living organisms with their own cell nucleus, evolved from symbioses of prokaryotic organisms (e.g., bacteria living in the cells or bodies of other organisms). The beginning of all living things, i.e., all microorganisms up to humans, can be traced back to an initial process of symbiosis and transformation of cells without a nucleus into the first eukaryotes about two billion years ago. Accordingly, the overriding principle of all evolution is *symbiotic cooperation*, from unicellular organisms to mitochondria to vertebrates: “I have already suggested that the eukaryotic cells characteristic of all forms of life arose by an evolutionary progress fundamentally different from that of discrete mutations” (Margulis, 1971, p. 55). The importance of symbiotic mechanisms in the evolution of all flora and fauna is increasingly recognized:

“Symbiosis is more than a biological curiosity, it is undoubtedly one of the most powerful drivers of evolution in the world of living things. [...] Thus the mechanisms of endosymbiosis, which renew the Darwinian view of evolution by descent with modification, in which one species is likely to give rise to two species, remind us that sometimes two species, once independent and recognizable, merge into one. Humans themselves can be viewed as an extremely integrated symbiotic community, consisting of eukaryotic cytoplasm and mitochondria, but also the archaea and bacteria that populate their gut microbiota, for example” (Selosse and Joyard, 2022).^4^

Endosymbiosis can be detrimental to the host (e.g., in bacterial diseases), but it is often a symbiotic relationship from which both sides benefit. The number of bacteria and living organisms in the human digestive system is greater than that of our own body cells. Without this symbiosis with many billions of microorganisms, humans could not live. Symbiosis is only one form of cooperation. Altruistic behavior has now been studied in many animal species and especially humans. This is where sociology, with its focus on social interdependence and social action, can make decisive contributions. What distinguishes humans from other animals is the ability to recognize ourselves as beings with self-awareness and the awareness that other people also have a self and can reflect about their self and that of others. What distinguishes us is the ability to cooperate by *understanding* others, knowing that they also perceive us in a way that we understand them. A distinctive feature of humans is their physiological and cognitive ability to speak and understand spoken language. Humans are also distinguished by their cognitive competence to recognize themselves as beings with an I and a Self, and to perceive that and how this works in other people as well. Language and other forms of communication also facilitate to pass on information and knowledge intergenerationally and in a differentiated explicit way.

In this respect, humans differ from other animals. A key aspect is the ability to understand oneself and the mental representations of the other. Animals, even very advanced primates, are inherently limited in their understanding of meaning. For nearly 30 years, Michael Tomasello compared the ontogenetic development of cognitive and cultural abilities in primates and humans. According to this, *shared intentionality* distinguishes every infant from all primates:

“Even very young children have a natural tendency to help other people solve their problems, even when the other person is a stranger and they get no benefit at all. However, our closest primate relatives also show some abilities in this direction, and this suggests that the common ancestor of chimpanzees and humans had a tendency to help even before humans began their unique path to hypercooperativeness” (Warneken and Tomasello, 2006, p. 1302).

According to this, only humans can develop *complex empathy in the sense* that they align their social behavior and actions not only with their own needs and preferences, but also with the (assumed) perceptions of reality and expectations of others, our interaction partners. In sociology, Symbolic Interactionism already emphasized the high importance of the meanings we produce and negotiate in social interactions. It assumes “that human beings act toward things on the basis of the meanings that the things have for them” (Blumer, 1969, p. 2). Things here refers to everything people perceive in their world, from physical objects to other people to ideas and theories. The corresponding meanings are thereby accessed, negotiated, and also changed in symbolic interactions. Similarly, but without any reference to this sociological knowledge that has been established for over a 100 years, Tomasello points out: “After the age of three, children begin to socially reflect on their communication efforts so that they appear intelligible and rational to others, and they begin to socially reflect on the impression they make on others in order to maintain their cooperative identity in the group” (Tomasello, 2019, p. 9).

Shared intentionality is thus practiced and trained from an early age in processes of symbolic interaction or, in a broader sense, *cooperation through understanding minds*. By the term *cooperation through understanding minds* we refer to the typically and exclusively human cooperation processes that are based on the assumptions that I understand my counterpart in his awareness and interpretation of a given situation and that my counterpart also assumes to understand my situational awareness. This is precisely what distinguishes humans from other animals; it is the capacity for this double reflection. Tomasello (2019, p. 18) summarizes, “The social outcome of early human adaptations to the need to forage together was a second-person morality: the tendency to relate to others in direct interaction, with a heightened sense of sympathy for (potential) partners and a sense of justice based on a genuine appreciation of self and others as equal partners in the cooperative enterprise.”

*Cooperation through understanding minds* does not always have to be free of conflict. It also includes conflicts due to varying interests, points of view or world interpretations. The evolutionary meaning of symbiosis and cooperation, however, contradicts the classical Darwinian and, above all, the Social Darwinian understanding according to which survival of the *fittest* primarily involves a constant competition as existence-destroying fighting between and within species. Such a narrative still forms the basis for nationalist, populist and racist ideologies. But also the assumption that it is a “biological commonplace that organisms evolve at one another's expense” (Pinker, 2010, p. 8993) still seems widespread. In contrast, recent research shows that, while violent conflict within and between social groups is a constant of human evolution, different social groups generally intermingled and interchanged goods and knowledge. Thus, among hunter-gatherer groups, any “mass capture [...] included thoughtful, pre-planned, cooperative preparation” (Scott, 2020, p. 78). In a more integrated perspective, *cooperation through understanding minds* could be considered a crucial element of ethodiversity that distinguishes the human species from others – without neglecting the overarching interrelations between behavioral dynamics at the levels of individuals, populations and species as an “intricate ensemble of abiotics, biotics, and artifacts which define the social niche” (Coca et al., 2021, p. 2; also Cordero-Rivera, 2017).

The first large state-like cities and later empires in Mesopotamia, Egypt, China, Greece, and Italy were more or less interested in dominating other groups and making them pay tribute, depending on the ecological and sociocultural conjuncture: “wars [were] more destructive than bloody” (Scott, 2020, p. 134, also 162f, 166–178). Scott emphasizes, however, that over millennia groups of people alternated between nomadic and sedentary ways of life (Scott, 2020, p. 218). The cities and empires that had been emerging, strengthening and decaying since ten thousand years were mainly interested in apprehending precious goods and captives as slaves during conquests and plunders, not in destroying them; “in any case, there is no evidence that people were slaughtered” (Scott, 2020, p. 221). Even where violent conquest was involved, it may have been an evolutionary advantage to be able to empathize with one's own people in understanding cooperation and with the groups to be fought in understanding competition. Evolution as a whole is a complex web of cooperation and competition. Classical Darwinism overemphasized the aspects of existence-destroying competition. These insights from evolutionary research are relevant to how humanity responds to the challenges of the Anthropocene.

## The great acceleration in the Anthropocene: Nature, culture and technology

**Figure 1:**
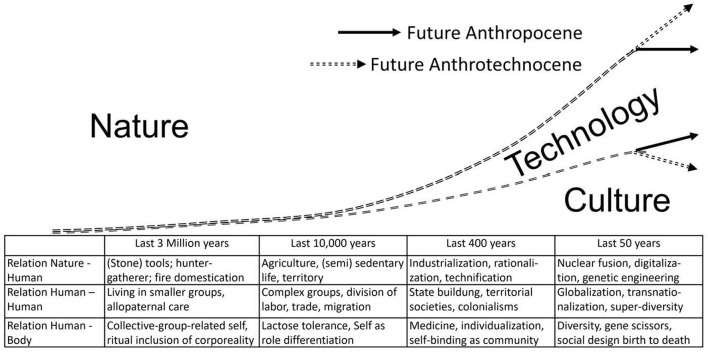
Development of the relation nature-culture-technology. Source: Author's own elaboration.

Our narratives about human evolution have a major impact on how we perceive the *great acceleration in the Anthropocene*. If we assumed that humanity evolved through random genetic mutations and selection of the fittest in the cutthroat struggle of individuals, groups and species, then a “carry on” of *trial and error* natural selection would be appropriate for meeting the challenges of the future. But if symbiotic relationships already existed at the beginning of the simplest living beings and if humans are characterized above all by shared intentionality and *cooperation through understanding minds*, then in the Anthropocene the forms of socio-cultural coexistence have to be reflected in a planetary perspective. This is not only about the relationship between nature and culture, but also about the significance of technology in the evolutionary process. Industrialization, digitalization and genetic engineering have had ambivalent effects on humans and the planet as a whole. In recent centuries, technology has come between humans and nature; it has become our second nature (Figure 1).

Roughly, four phases of human development can be distinguished. The last three million years refer to the emergence of *Homo sapiens* since the Stone Age. The last 10–12 thousand years represent (transition to) sedentary, agrarian life, beginning in Mesopotamia and Asia. The last 400 years mark the period of industrialization, and the last 70 years can be described as the beginning of the Anthropocene. The vertical axis of Figure 1 represents the evolution from the dominance of nature to the growth of the share of culture and technology. The horizontal axis represents evolutionary periods, but in a non-metric scale. Technology is an expression of human cultural creation and at the same time increasingly appears as part of the natural environment. This is true of roads, buildings, manufacturing plants, modern agriculture, hospitals, the Internet, and social media. The intergenerational transmission of technology takes place, on the one hand, through cultural learning in schools, universities, etc., and, on the other hand, through the silent compulsion of the quasi-natural artifact relations as a result of technical niche production.

In the first two stages - the Stone Age and sedentary agrarian life - human life was almost completely permeated and determined by nature. Culture gradually gained weight as the ability to domesticate fire, use tools, keep domestic animals, and sow and harvest. It is only in the third period, industrialization, that the living world is largely structured by human-made artifacts. For our daily life, the part of culture and technology becomes more and more important, and the dividing lines between nature, culture and technology become more and more permeable. This development is not linear, but asymptotic, as can be seen from the short time periods of the last two stages of development in relation to the preceding ones. There is much to suggest that the dynamics of technical development are becoming increasingly decoupled from our sociocultural abilities to master them. Technology is spreading mainly by liberal-market mechanisms, mainly without socially consented goals and applications.

An extensive study has documented the socioeconomic and technological developments of the last three centuries (Steffen et al., 2015). In this context, the following twelve indicators show an *exponential* increase since the mid-20th century: world population, real gross domestic product, foreign direct investment, urban population, primary energy consumption, fertilizer consumption, construction of large dams, water consumption, paper production, transport, telecommunications and international tourism. The impressive acceleration since the 1950's in so many dimensions underscores the challenges of the Anthropocene, which requires human responses (in the dual sense of human responses and humane, sustainable sociocultural responses).

Concerning the acceleration of both, the degree of human intervention in planetary mechanisms and the speed of fundamental shifts in ecologic and sociocultural environments of human coexistence, in an evolutionary perspective there arise fundamental challenges. Meanwhile some biologically based human traits–like physical fitness or cognitive capacities of the human brain–developed over hundreds of thousands of years by intertwined processes of niche production, adaption and selection, the human biophysical condition had little chance to develop in step with the (mainly human-originated) shifts in environmental living conditions. (Odling et al., 2003, p. 261) take the example of population concentration and epidemics, as “the construction of villages, towns, and cities creates new health hazards associated with large-scale human aggregation, such as the spread of epidemics.” COVID-19 and the global spread of its variants due to the degree of globalization and transnationalization are an illustration. The authors argue that this could lead to (biological) selection of more resistant genotypes or/and to cultural development of vaccines and improved health infrastructure and assume (Odling et al., 2003): “As cultural niche construction typically offers a more immediate solution to new challenges, we anticipate that cultural niche construction will usually favor further counteractive cultural niche construction, rather than genetic change.” COVID-19 caused fundamental debates in an evolutionary perspective and revealed the challenges of dealing with men-made accelerations in shifting human conditions and the necessity to find humane solutions.^5^

## Conclusions: What kind of Anthropocene do we want?

With regard to the Anthropocene, there are several focal points of debate in an evolutionary-historical perspective. One is the time line of the Anthropocene in evolutionary perspective: Has the fate of the planet really only been decisively shaped by humans since about the middle of the 20th century? Or does the Anthropocene already begin with the sedentary life of *Homo sapiens* or even the domestication of fire (Scott, 2020, p. 52ff)? A second discussion concerns the options and directions of possible human intervention. Here, there are contrasting positions on the role of technology and of culture in addressing current challenges. At one extreme is the vision of an Anthrotechnocene in which the major challenges are to be solved primarily through the (further) expansion of technologies. At the other extreme is the position of a planetary-human Anthropocene in which culture, cooperation through understanding minds, and social institutions are central. For these debates, we should not limit ourselves to analyzing the last 70 years (of globalization and accelerated digitalization), nor the last 400 years (of industrialization).

With regard to social change, diagnoses of comparatively short time windows of a few decades or centuries have been predominant in sociology so far. This is true for work on the Second or Reflexive Modernity Age (Beck et al., 1994), but also for studies on the varieties of capitalisms (Hall and Soskice, 2001; Bizberg, 2019). In order to include a developmental perspective on the Anthropocene, sociology should seek greater dialogue with other disciplines. Richerson and Boyd argue for a cultural turn in biology and for an evolutionary opening of the culture-oriented sciences because “nothing about culture makes sense except in light of evolution” (Richerson and Boyd, 2005, p. 237). Sociology should also focus more on the relationship between nature, culture, and technology in *the long run*, in the sense of deep history (Scott, 2020).

In this context, the gradual or abrupt shift of the relationship between nature, culture and technology in evolution comes into focus. Sociology can help to reintegrate technology development more strongly into cultural-institutional governance. We are witnessing unchecked digitization that is barely channeled through social norms and institutions, such as social media that are not subject to legal or professional codes similar to print or TV media. Instead of taking into account the increasing social inequality and exploitation of the planet, some multi-billionaires are already discovering space as their techno-fixated exit strategy to escape the planet's problems. Visions of a anthrotechnocene or capitalocene can be identified here, in which socioculturally sustainable changes are not sought, but rather technology-determined, socially discriminatory solutions according to the principle of “survival of the most brutal” (Moore, 2016; Adloff and Neckel, 2020).

If specifically human capabilities have emerged by cooperation through understanding individual and collective minds, then the challenges of the Anthropocene should also be addressed through it. It is a matter of developing globally coordinated cooperation at the levels of civil society (profit and non-profit), organizations and the international community of states. This may seem illusory, but it is more realistic and sustainable than relying on technology and (further) domination of nature. Above all, sociology can contribute to developing a planetary-human multi-level governance of *social institutions* for the 21st century. One of the core competencies of sociology is the analysis of social institutions as programs of action (laws/rules, norms, cognitive maps) that structure specific areas of social life, are inherited socioculturally and offer accountability in the complexity of human life.

Such institutions structure social coexistence at the local, national, supranational, global and transnational levels. They emerge and stabilize through mechanisms of habitualization, explication, typification, and formalization. Institutions provide social identity, stability, integration, and predictability. Examples are forms of greeting, forms of living together in families and networks, educational systems, bundling of work activities in occupations and professions, regulation of conflicts at the workplace through works councils, rule of law, establishment of agreements and cooperation through representative democracy, international law, human rights. A major challenge in the Anthropocene is the development of social institutions capable of structuring cooperation through understanding minds in an increasingly complex and diverse world. Technology can support this, but not replace it.

It would be worthwhile to analyze the formation, stabilization and possible weakening of social institutions in an evolutionary-historical and global perspective in a comparative and multidimensional way. This would be as interesting for the Chinese *guanxi* as for the Indonesian *lumbung*, African ubuntu, the Central European *commons* or the Mexican *compadrazgo*. Institutionally, the European Union (EU) is also interesting in the Anthropocene because it represents innovative ways of coordinating human coexistence in very large and complex interdependent contexts. It has incorporated fundamental ideas from the French and many other civil revolutions into its *acquis communautaire*, which is something like its DNA.^6^ Over many decades, it has undergone a remarkable evolution toward a sociocultural community in diversity. Although the EU member states still have divergent interests, although they have done little to come to terms with their war crimes and colonial pasts, although there are large gaps between their declamations to be a “community of values” and their realpolitik, for example with regard to the adequate protection of refugees, there are hardly any comparable *cross-state* governance initiatives. Beyond Eurocentrism, the project of combining social market economy with welfare state, democracy with efficiency, socio-cultural diversity with sustainable economy, unification with decentralization seems promising (Beck and Grande, 2004).

Sociology's reservations about evolutionary research and theory are understandable. However, in view of the enormously expanded empirical findings and theoretical discussions in biology, geology, anthropology, and the behavioral sciences, they hinder the further development of their own discipline and withhold from evolutionary research the substantial contributions that sociology can add (Turner and Maryanski, 2016; Baldus, 2017). In the Anthropocene, present-tense diagnoses over short-time spans are not enough. For sociology, it would be promising to deal more intensively with evolutionary trajectories of the relationship between nature, culture, and technology and analyze social action, social orders, and social change in an explicitly long-term evolutionary perspective (Hodder, 2020).

## Data availability statement

The original contributions presented in the study are included in the article/supplementary material, further inquiries can be directed to the corresponding author.

## Author contributions

The author confirms being the sole contributor of this work and has approved it for publication.

## Footnotes

^1^For the thesis of selfish genes see Dawkins (1976); the interrelation between genes, minds, groups and culture is a central topic of scientific debates, where most biologists mainly assume a “nested hierarchy of units” of selection from genes over individuals up to (socio-cultural) groups (e.g. Wilson and Sober, 1994; Wilson, 1999, e.g. p. 148 and 179). As an example, Paolilli (2011) makes an argument that altruism could lead to group selection, and not only the other way round.

^2^For reasons of sociology's neglect of evolutionary science see also Takács (2018) and Chattoe-Brown (2019).

^3^For the timing of human development in relation to climate change, see, for example, Krause and Trappe (2021) and https://humanorigins.si.edu/evidence/human-evolution-interactive-timeline.

^4^For symbioses in flora, e.g., between lichens and fungi in the evolution of the planet, e.g., Nelsen et al. (2020).

^5^See e.g. Blühdorn et al. (2021) and contributions on https://thisviewoflife.com/gene-culture-and-potential-culture-gene-coevolution-the-future-of-covid-19/.

^6^See https://eur-lex.europa.eu/summary/glossary/acquis.html?locale=de.
